# Assessing horizontal gene transfer in the rhizosphere of *Brachypodium distachyon* using fabricated ecosystems (EcoFABs)

**DOI:** 10.1128/aem.01505-24

**Published:** 2024-11-04

**Authors:** Shweta Priya, Silvia Rossbach, Thomas Eng, Hsiao-Han Lin, Peter F. Andeer, Jenny C. Mortimer, Trent R. Northen, Aindrila Mukhopadhyay

**Affiliations:** 1 Biological Systems and Engineering Division, Lawrence Berkeley National Laboratory, Berkeley, California, USA; 2 Department of Biological Sciences, Western Michigan University, Kalamazoo, Michigan, USA; 3 Environmental Genomics and Systems Biology Division, Lawrence Berkeley National Laboratory, Berkeley, California, USA; 4 School of Agriculture, Food and Wine, University of Adelaide, Adelaide, South Australia, Australia; Shanghai Jiao Tong University, Shanghai, China

**Keywords:** EcoFAB, HGT, rhizosphere, *Brachypodium*, conjugation, triparental, frequency, *Pseudomonas putida*, mesocosm, salinity, *Burkholderia* sp.

## Abstract

Horizontal gene transfer (HGT) is a major process by which genes are transferred between microbes in the rhizosphere. However, examining HGT remains challenging due to the complexity of mimicking conditions within the rhizosphere. Fabricated ecosystems (EcoFABs) have been used to investigate several complex processes in plant-associated environments. Here we show that EcoFABs are efficient tools to examine and measure HGT frequency in the rhizosphere. We provide the first demonstration of gene transfer via a triparental conjugation system in the *Brachypodium distachyon* rhizosphere in an EcoFAB using *Pseudomonas putida* KT2440 as both donor and recipient bacterial strain with the donor containing a mobilizable and non-self-transmissible plasmid. We observed that the frequency of plasmid transfer in the rhizosphere is potentially dependent on the plant developmental stage and the composition and amount of root exudates. The frequency of plasmid transfer also increased with higher numbers of donor cells. We demonstrate the transfer of plasmid from *P. putida* to another *B. distachyon* root colonizer, *Burkholderia* sp. OAS925, showing HGT within a rhizosphere microbial community. Environmental stresses also influenced the rate and efficiency of HGT in the rhizosphere between different species and genera. This study provides a robust workflow to evaluate transfer of engineered plasmids in the rhizosphere when such plasmids are potentially introduced in a field or other plant-associated environments.

**IMPORTANCE:**

We report the use of EcoFABs to investigate the HGT process in a rhizosphere environment. It highlights the potential of EcoFABs in recapitulating the dynamic rhizosphere conditions as well as their versatility in studying plant-microbe interactions. This study also emphasizes the importance of studying the parameters impacting the HGT frequency. Several factors such as plant developmental stages, nutrient conditions, number of donor cells, and environmental stresses influence gene transfer within the rhizosphere microbial community. This study paves the way for future investigations into understanding the fate and movement of engineered plasmids in a field environment.

## INTRODUCTION

Horizontal gene transfer (HGT) enables transmission of genetic material between otherwise unrelated organisms via transformation, transduction, or conjugation. This process allows an organism to acquire new functions that can shape bacterial evolution or fitness by providing new genes that would be unlikely to arise from simpler spontaneous mutations. HGT can lead to the appearance of multiple identical, redundant operons within the soil communities which promote intra- and interspecies variability (1, 2). Conjugation is one of the major HGT processes by which bacteria acquire DNA and new functionality to adapt and overcome environmental stresses and compete in their ecological niches (3). Non-conjugative plasmids from donor cells can be mobilized to recipient cells by helper plasmids; this process is known as triparental conjugation (4).

**Fig 1:**
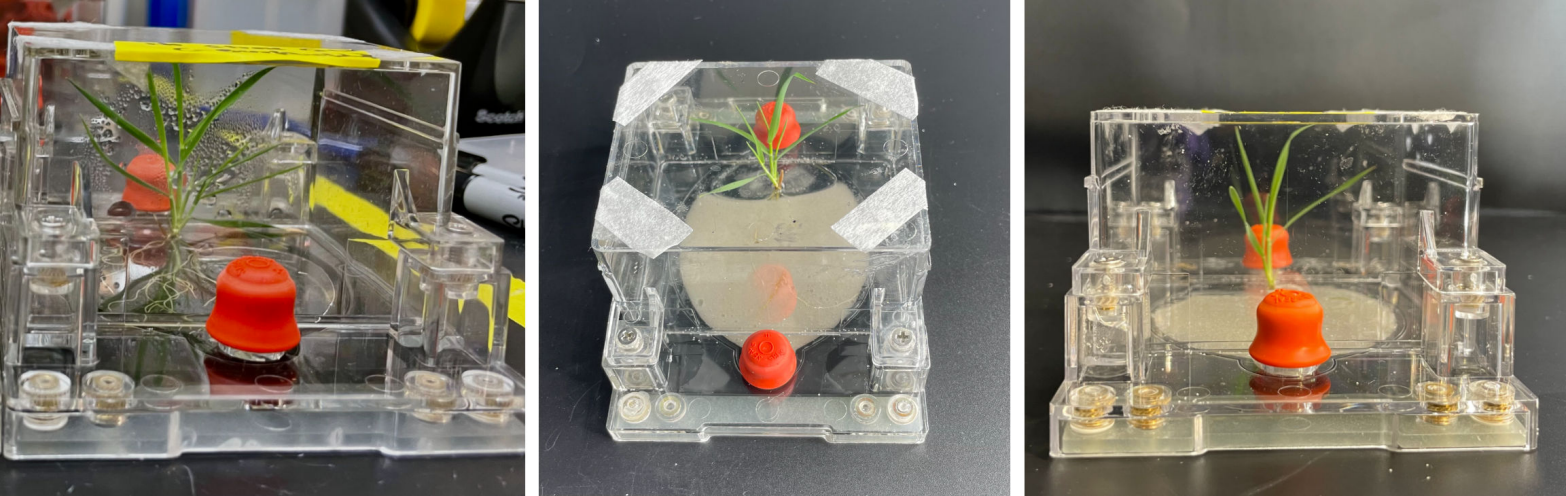
*Brachypodium distachyon* grown in EcoFAB 2.0 (microcosms used in this study). Pictures of EcoFABs taken with liquid medium and sand from above and the side, respectively (left to right).

Gene transfer phenomena are important to examine but are less understood in the context of genetically modified microbes and their potential use or release into the environment (2). Specifically, HGT remains challenging to study and quantify in natural environments like the rhizosphere or in the soil matrix. Recently, lab-scale microcosms such as rhizotrons, rhizobox, or nylon soil pouches have been designed that allow continuous monitoring throughout the plant developmental stages and non-destructive sampling of the rhizosphere or root metabolites in real time (5). Of these, fabricated ecosystems (EcoFABs) are open-source three-dimensional printable chambers that are highly standardized reproducible tools enabling mechanistic studies of plant-microbe interactions under controlled laboratory conditions (Fig. 1) (6, 7). Several complex processes occurring in the rhizosphere under natural environmental conditions have been simulated and studied in EcoFABs (6, 8, 9). In such studies, repeatability is critical. Sasse et al. (8) found that *Brachypodium distachyon* growth in EcoFABs was reproducible across four laboratories for a number of morphological and metabolic traits of root tissue and root exudates (REs) (8). EcoFABs may also enable measurement of gene transfer frequency under controlled conditions and mimic key aspects of the natural rhizosphere environment (7).

The frequency of gene transfer and uptake is potentially enhanced by availability of nutrients in the rhizosphere from root exudates, and other favorable conditions such as moisture and root surface that make the rhizosphere a preferred site for microbial colonization (10). The high cell densities and metabolic activity of microbes in the rhizosphere may also influence the HGT processes (11, 12). In addition to nutrient content, environmental stresses such as drought or salinity could also be the driving conditions for rhizosphere bacteria to acquire genes and plasmids from others in the microbial community to compete and survive (13). To our knowledge, there are few studies that have investigated the impact of abiotic stress conditions on the frequency of HGT; however, this could also be a critical parameter influencing this phenomenon (14, 15). For instance, studies have reported that the rate of conjugal transfer of plasmids is influenced by changes in soil physico-chemical conditions such as moisture, osmotic stress, pH, temperature, or soil components. This indicates that changes in rhizosphere conditions due to these abiotic stresses could also impact the HGT frequency (16, 17). While some studies have reported possible transmission of plasmids between bacteria of distant genera (16, 18), this process is yet to be investigated in detail in the rhizosphere environment.

Here, we developed a model system with established HGT efficiencies to monitor plasmid uptake by root-associated bacteria in the EcoFAB root chambers and to measure HGT. We describe a triparental conjugation system in the presence of *B. distachyon* roots. Establishing this baseline allowed assessment of other parameters such as donor-to-recipient ratio and environmental stresses that may impact the conjugation frequency (CF) in the rhizosphere. Furthermore, we also examined intergeneric plasmid transfer using a non-model bacterium of the rhizosphere microbial community as a recipient. To understand the possibility of transfer of a mobilizable and non-self-transmissible plasmid without a helper strain, we have also tested a biparental mating system under routine laboratory or environmentally stressed conditions.

## RESULTS AND DISCUSSION

### Detection and quantification of horizontal gene transfer via conjugation in the rhizosphere of *B. distachyon*

**Fig 2:**
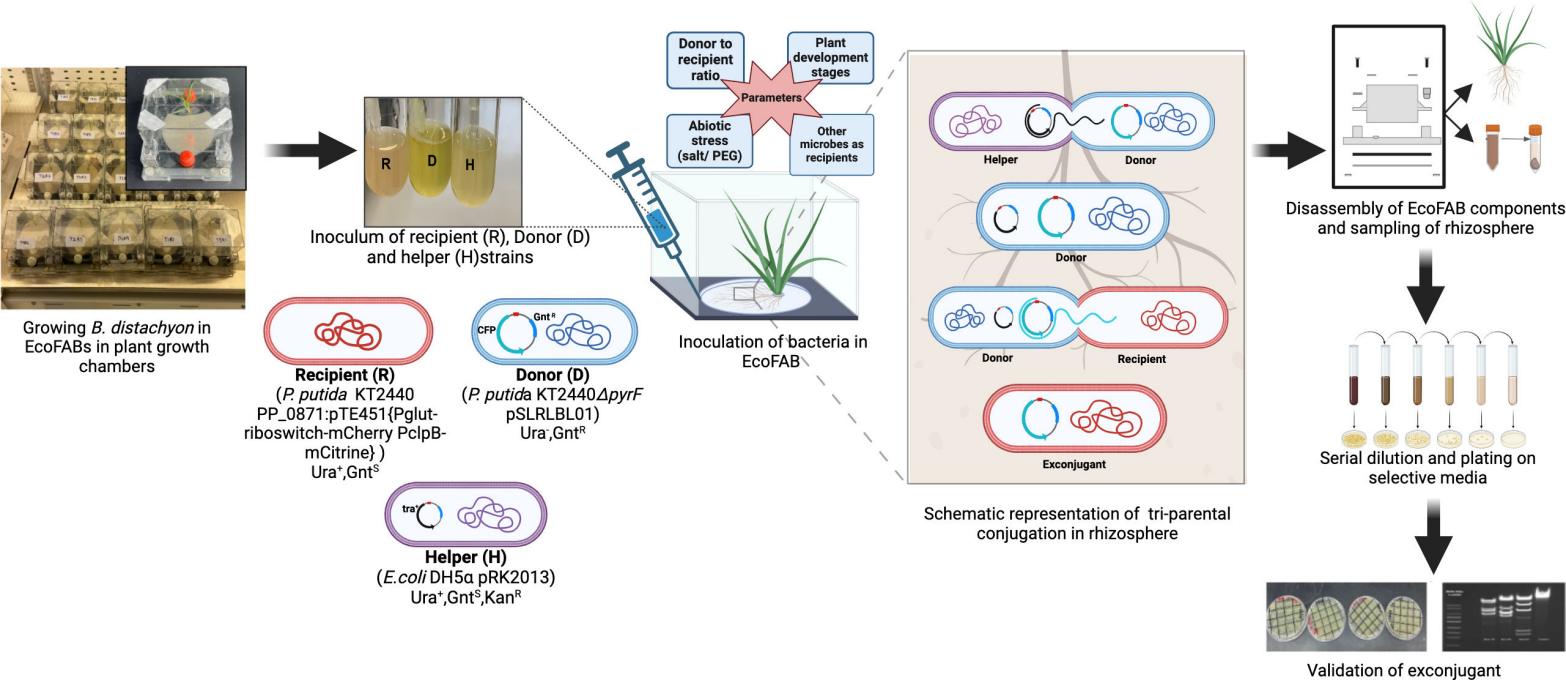
Experimental workflow for quantification of conjugation frequency in the rhizosphere of *B. distachyon* grown in EcoFABs. (Left to right) Growing *B. distachyon* in EcoFABs in the plant growth chamber for 2 weeks followed by inoculation of donor (D), recipient (R), and helper (H) strains in the rhizosphere ; schematic representation of triparental conjugation; sampling of rhizosphere 24 h post-inoculation, by dissolving it in 5-mL phosphate-buffered saline, and vortexing for 5 min (1mL aliquot was collected after sand settled, followed by serial dilution and plating of 100-µL aliquot on selective media); validation of exconjugants by streaking on selective media and restriction enzyme digestion of isolated donor plasmid from exconjugants. This figure was created with biorender.com. PEG, polyethylene glycol.

**Fig 3:**
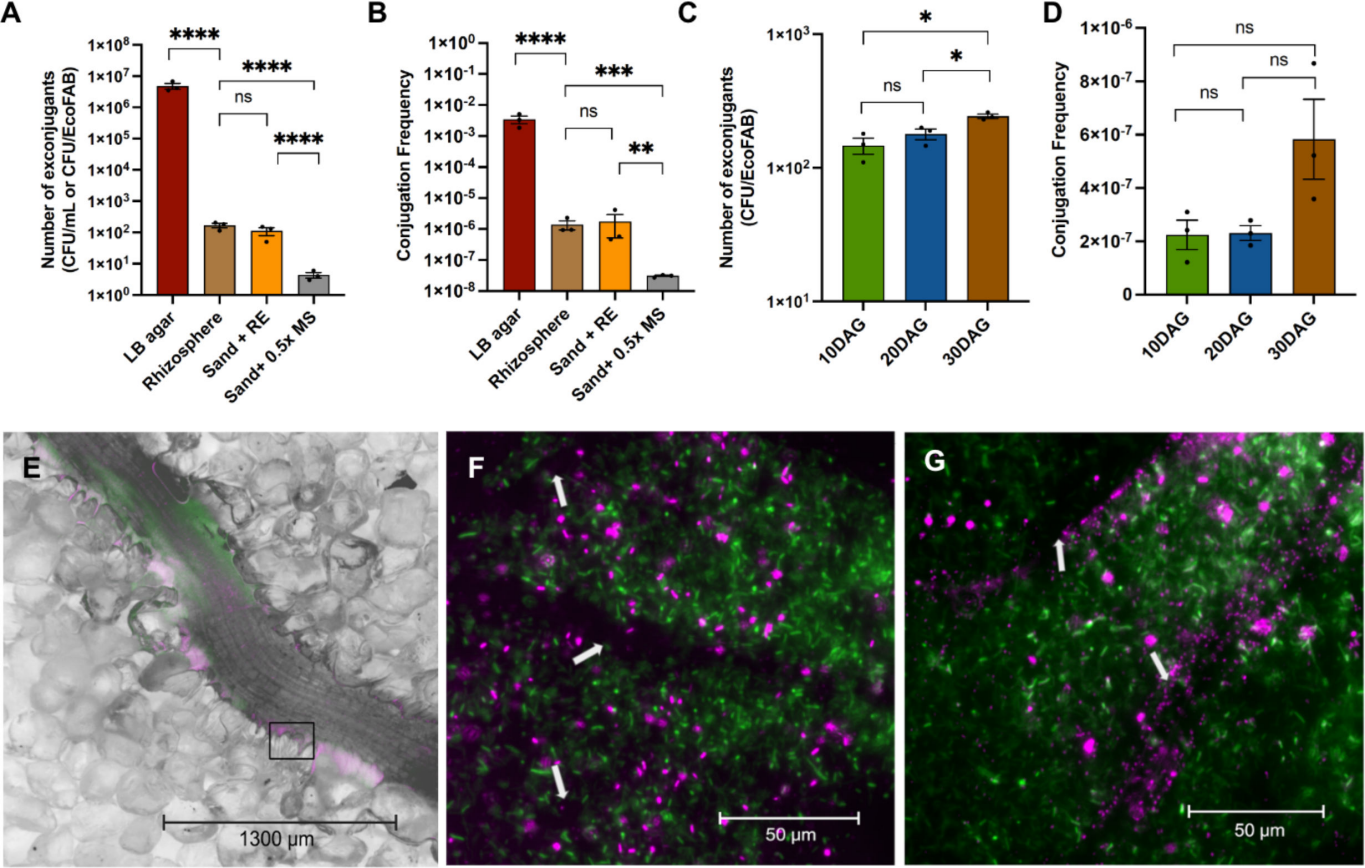
The number of exconjugants (CFU/mL for LB agar or CFU/EcoFAB). CFU counts were normalized to the actual volume of media used per experiment (**A**) and the conjugation frequency (**B**) observed with different treatments. Conjugation frequency observed at different developmental stages of the plant (**C**). The error bars show the mean standard error of three replicates. Microscopic images of *B. distachyon* roots inoculated with proxy donor (green) and recipient (magenta) strains in EcoFABs at using ×2 (**D**) and ×40 (**E and F**) objectives. The black box in panel E represents the area magnified for panels F and G, and the white arrows in panels F and G represent the root hair. The mCherry channel is false-colored magenta to indicate the location of recipient strains in this two-channel composite photomicrograph. DAG, days after germination; ns, non-significance; RE, root exudate.

Triparental mating under laboratory conditions has been reported for many Gram-negative bacteria and served to develop a model system for our study. We initiated this project by first demonstrating the desired HGT system with *P. putida* and selection for growth with an antibiotic marker that would enable detectable plasmid transfer events under optimal conditions. This workflow is described in Fig. 2. The recipient *P. putida* strain would grow in the presence of gentamicin if it acquired a plasmid harboring an *aacII* gene (conferring gentamicin resistance) from the donor strain via the triparental conjugation. Moreover, the recipient with the donor plasmid would fluoresce in the presence of arabinose due to the expression of the *cfp* gene. This experimental setup allowed us to counterselect against the donor strain and to visualize any gene transfer based on fluorescence. We have also used a helper strain that harbors the pRK2013 that has been widely used as a conjugative helper plasmid to assist the transfer of mobilizable and non-self-transmissible plasmids from one bacteria to another (19, 20). All three strains (donor, recipient, and helper) were spotted on a solid agar plate supplemented with Luria-Bertani (LB) medium and 25-µg/mL uracil. If the recipient strain successfully acquired the plasmid from the donor strain, we could select for this event by plating the mixture onto M9 glucose plates with gentamicin. The *P. putida* donor strain has the ∆*pyrF* deletion and is unable to grow in minimal medium without uracil supplementation, which we use as a counterselection against the donor strain in calculating HGT events. The total number of exconjugants observed was 4.8 × 10^6^ CFU/mL with a conjugation frequency of 3 × 10^−3^ (coefficient of variation = 47%; Fig. 3A and B). To further demonstrate HGT in the rhizosphere, we inoculated the three strains in the rhizosphere of *B. distachyon* grown in EcoFABs and plated the conjugants after 24 h. In the *B. distachyon* rhizosphere, the total number of exconjugants observed were 167 CFUs per EcoFAB with a conjugation frequency of 1.4 × 10^−6^ (coefficient of variation = 56%; Fig. 3A and B).

To our knowledge, this is the first demonstration of HGT using an EcoFAB. The frequency of HGT in the rhizosphere in EcoFABs was lower than that on agar plates, potentially due to fluctuating nutrient conditions of the rhizosphere (21). The number of exconjugants and conjugation frequency in the rhizosphere observed in this study were similar or higher than those found in other studies (22). Several studies have previously confirmed HGT in soil under various conditions using different soil microcosms without plants (23–26). Nevertheless, there are very few studies in recent years that have shown HGT in rhizospheres using lab-scale microcosms due to the complexity of recapitulating dynamic conditions of a plant-associated environment as well as the risks associated with using engineered systems (10, 27). Our results provide evidence for the great potential of EcoFABs as a reliable and consistent platform to examine the fate and migration of target plasmids or genes, including those from engineered systems, in plant-associated environments.

### Impact of root exudates and plant developmental stages on frequency of HGT in the rhizosphere

We examined the influence of the presence of root exudates on the conjugation frequency in the rhizosphere by comparing the conjugation frequency in the rhizosphere (with a plant) to that of EcoFABs filled with sand only [sand + 0.5× Murashige and Skoog (MS)]. We also tested if removal of the plant root influenced this process when the root exudate was still present (sand + RE) to understand if the presence of root surface is an essential requirement for the HGT process in the rhizosphere. The procedure of removing the plants and adding back the root exudates in the sand filled EcoFABs is described in detail below.

The conjugation frequency decreased substantially (~40×) in the EcoFABs with sand only (sand + 0.5× MS) compared to the rhizosphere (Fig. 3A and B). This indicated that the compositional change in the rhizosphere due to the presence of plant root exudates could be a major facilitator of HGT in the rhizosphere environment. However, we observed no substantial difference in the conjugation frequency between the rhizosphere and the EcoFABs filled with sand amended with root exudates (sand + RE), which indicates that the root exudate is a major requirement for uptake of plasmids, while presence of the plant root surface may not be an essential factor determining the HGT frequency. Root exudates provide the necessary nutrients for improved growth and microbial activity, which may also stimulate the uptake and conjugal transfer of the plasmid (11, 22, 28). Additionally, we did not find any major differences in the bacterial density under these two conditions (rhizosphere and sand + RE), while the density of donor bacteria was several times lower in the EcoFABs containing only 0.5× MS medium (Fig. S1). The microscopy images also suggest that the bacteria (both donor and recipient) are primarily present in the vicinity of roots and root hairs where most of the root exudates are released (Fig. 3E through G). These results suggest that the root exudates could be an important criterion for determining the HGT frequency mainly by influencing the bacterial growth and survival in the rhizosphere. Mølbak et al. (10) reported that the amount of root exudates and root growth are the key parameters impacting plasmid transfer in the rhizosphere, while the root surface features did not play an important role in this process. They compared the rates of horizontal plasmid transfer in the rhizosphere of pea and barley and showed that pea roots had 10 times higher transfer rate due to higher rate of root exudation of pea roots, which increased the colonization of donor strains (10).

We also examined the impact of different plant developmental stages on the conjugation frequencies as it may impact the amount and composition of the root exudates (29). The results showed that the number of exconjugants increased by ~1.5×, and the HGT frequency increased by ~3× in the later stages of the plants [30 days after germination (DAG)] compared to early phases of growth (Fig. 3C and D). These slight changes could be due to higher amounts of total root exudates in the plants at later developmental stages, leading to higher root colonization by donor bacteria and thus stimulating the HGT (Fig. S2) (10, 11, 28). To understand the changes in the composition of carbon substrates in root exudates, we analyzed the composition of organic acids and sugars possibly present in the root exudates of *Brachypodium* at the three stages of the plant (30). The total amount of organic acids detected (malic acid, citric acid, and succinic acid) was higher than that of sugars (glucose and xylose) at all stages of plant growth up to 30 DAG (Fig. S3). Several studies have confirmed that organic acids in the plant root exudates play a major role in inducing chemotaxis and therefore are crucial for root colonization by bacteria in the rhizosphere (31–33). In our study, the concentration of one specific organic acid (malic acid) increased proportionally with the plant age and could be correlated with the increased number of exconjugants and conjugation frequency at 30 DAG (Fig. S3). Similar results were reported in a study which showed that certain organic acids from plant root exudates stimulated the natural transformation of *Acinetobacter* sp. BD413 with the plasmid pFG4 in soil (34). Nevertheless, additional direct testing of these metabolites is required to completely understand the impacts of different root exudate components on HGT efficiency in the rhizosphere.

The HGT frequencies at all time points in Fig. 3B were lower than that of the 14 DAG plants shown in Fig. 3A. This could be due to better growth of recipients in the set of plants grown for determining the impact of developmental stages on conjugation frequencies.

### Measuring impact of donor-to-recipient ratio on conjugation frequency in the rhizosphere

**Fig 4:**
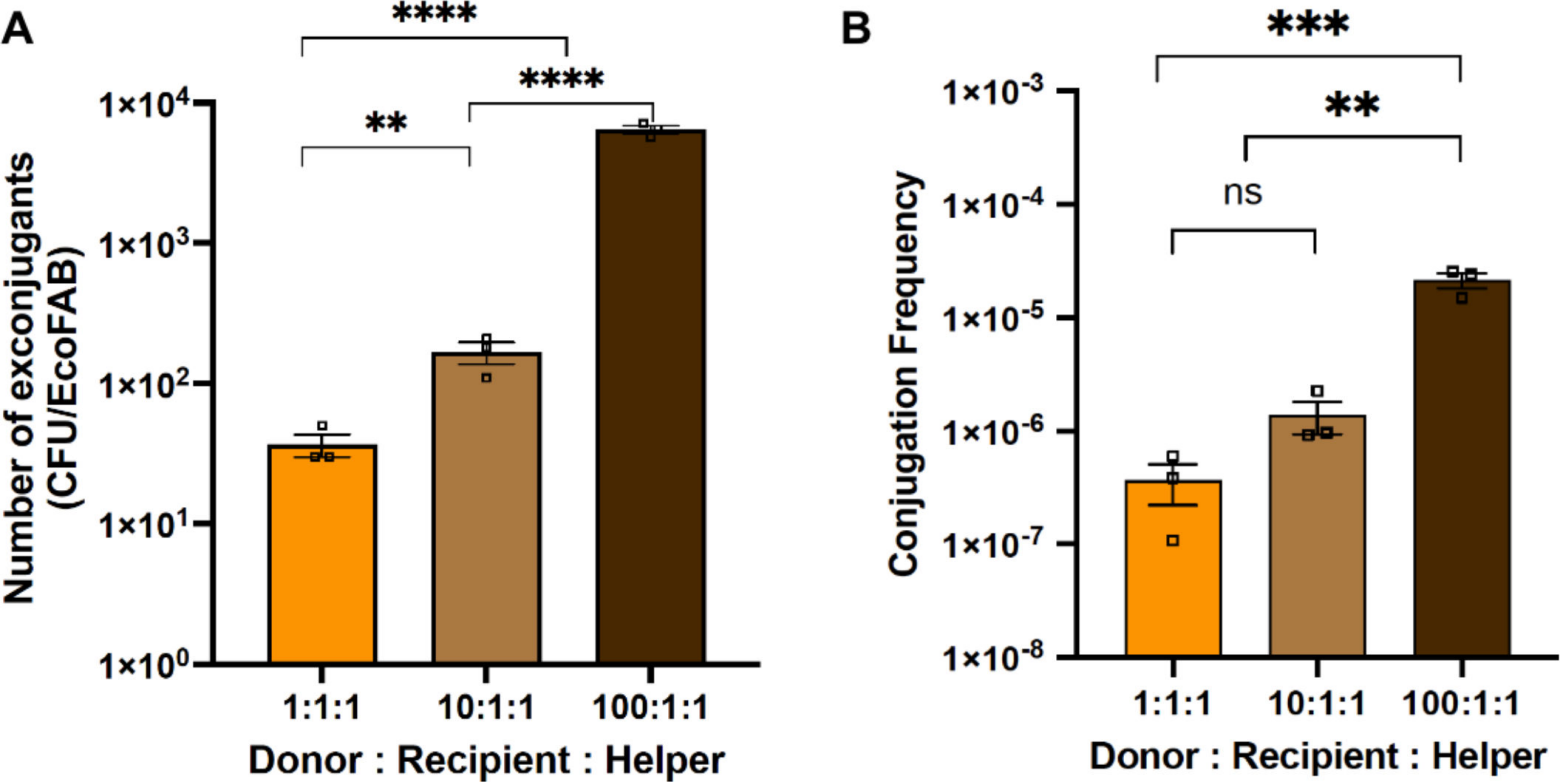
Change in the number of exconjugants (CFU per EcoFAB) (**A**) and the conjugation frequency (**B**) with increase in the donor-to-recipient-to-helper ratio. The error bars show the mean standard error of the three replicates.

HGT via conjugation requires physical contact between donor and recipient. Therefore, the ratio of donor to recipient (RD:R) cells has been identified as one of the most critical factors that determine the frequency of conjugal transfer in several studies (17, 35). To understand the impact of inoculum size of the donor strain on conjugation frequency in the rhizosphere, we used three different ratios of donor to recipient to helper: 1:1:1, 10:1:1, and 100:1:1. Results showed that increasing the inoculum size of the donor by 10× or 100× significantly improved the conjugation frequency in the rhizosphere by ~4× and ~60×, respectively (Fig. 4). Lampkowska et al. (35) published a standardized and optimized protocol for conjugation between two lactococcal strains and found that the frequency increased by increasing the ratio from 1:10 to 1:1 (35). However, in a recent study, Shi et al. (17) reported that the smaller the RD:R, the higher the exconjugant numbers (17). It is therefore possible that the impact of RD:R is variable under different experimental conditions and may also depend on the colonization rate or fitness of the two bacterial strains in the rhizosphere environment. In the case examined in the present study, the final donor strain counts were generally lower than the recipient strain under all conditions (Fig. S1, S2, and S4) possibly due to its dependency on the presence of uracil for survival, and therefore, increasing the ratio of RD:R improved the conjugation efficiency.

### Influence of environmental stress on the conjugation frequency in the rhizosphere

**Fig 5:**
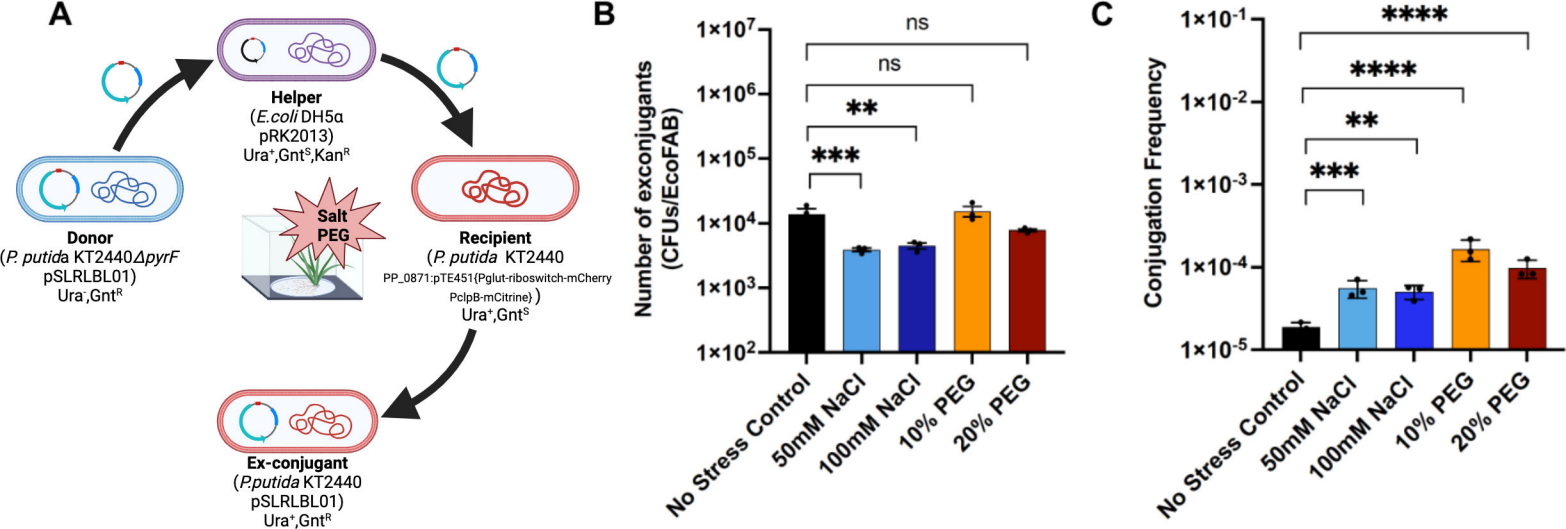
Schematic of conjugation experiment in EcoFABs (**A**), the number of exconjugants (CFU per EcoFAB) (**B**) and the conjugation frequency (**C**) observed under salinity and osmotic stress conditions. The error bars show the mean standard error of the three replicates.

Although there are few studies that have tested the impact of rhizosphere environmental conditions on HGT frequencies, two studies have shown that physical conditions of soil such as soil pH, moisture level, soil type, or the mineral composition have an influence on the transfer of plasmids (16, 17). Drought and other associated changes in the rhizosphere could be another important parameter that influences HGT frequency; however, it has not yet been investigated in detail. Osmotic changes can also be exogenously induced using polyethylene glycol (PEG) and were implemented using 10% and 20% PEG as described in Materials and Methods. To evaluate the impact of salinity-based stress conditions on HGT in the rhizosphere, we also added salt at 100- and 200-mM concentrations into the EcoFABs during the seedling transfer stage (see “Detection and quantification of horizontal gene transfer via conjugation in the rhizosphere of *B. distachyon*,” above) (Fig. 5A). Results indicate that the number of exconjugants was highest in the rhizosphere with 10% PEG with ~1.5 × 10^4^ CFUs/EcoFAB, while higher concentrations of PEG (20%) decreased the number of exconjugants compared to the no-stress controls. Addition of salt to the rhizosphere decreased the number of conjugants fourfold compared to the rhizosphere with no stress compounds (Fig. 5B). The conjugation frequency increased significantly (*P* ≤ 0.05) under all stress conditions compared to the no-stress controls and was overall higher in PEG than salt (Fig. 5C).

Our results show that the HGT frequency may vary with the environmental conditions in the rhizosphere; it was found to be increased if the plant is exposed to a moderate (sub-inhibitory) level of salinity/osmotic stress. It was previously reported that high levels of stress reduce the HGT frequency due to the inhibition of cell activities. Contrary to this, sub-inhibitory levels of stress might activate the global stress response mechanisms in bacterial cells, e.g., biofilm formation or increased membrane permeability, creating favorable conditions for HGT in the rhizosphere (36, 37). The concentrations of PEG or salt used in this study were sub-inhibitory (not lethal) as the fitness of both recipient and donor decreased as compared to no-stress controls (Fig. S4) and therefore could be the reason for higher HGT frequencies. This is consistent with the report by de la Rosa et al. (38), in which sub-inhibitory levels of antibiotics were found to increase the conjugation frequency due to activation of SOS response genes (38). Also, the lower number of recipient strains under all conditions compared to no-stress controls could have resulted in higher HGT frequencies (NC/NR). This suggests that the recipient bacteria may have a higher plasmid uptake efficiency via conjugation on exposure to stress as observed in some previous studies (39, 40). The lower number of recipients could also be related to the change in composition of plant root exudates due to a stressful environment that may impact the colonization and fitness of microbes in the rhizosphere (41, 42). However, it is not yet confirmed if such compositional change could directly influence the HGT frequencies in the rhizosphere and needs further investigation. Additionally, it is also important to note that the impact of stress on the number of donor cells is less substantial than that of the recipient cells and could be due to the presence of a plasmid that may provide a fitness advantage to their hosts under certain conditions in plant-associated environments (43).

Thus, several factors in the soil or rhizosphere may influence the efficiency with which microbes take up plasmids. However, it is not certain at this point whether this is due to changes in plant root exudate compositions or the direct impact of the stress on conjugation efficiency of donor/recipient or a combined influence of both. As such, it is important to consider multiple parameters while investigating the fate of plasmids or genes intentionally or unintentionally disseminated into a field and/or plant-associated environment.

### Detection of HGT between bacteria of different genera in the rhizosphere

To determine whether the plasmid of one bacterial species in the rhizosphere is also taken up by bacteria of other species or genera, we performed the HGT experiments in the EcoFABs with the plant-associated bacterium *Burkholderia* sp. OAS925, which has been shown previously to be an efficient colonizer of the *Brachypodium* rhizosphere (9), as the recipient strain.

**Fig 6:**
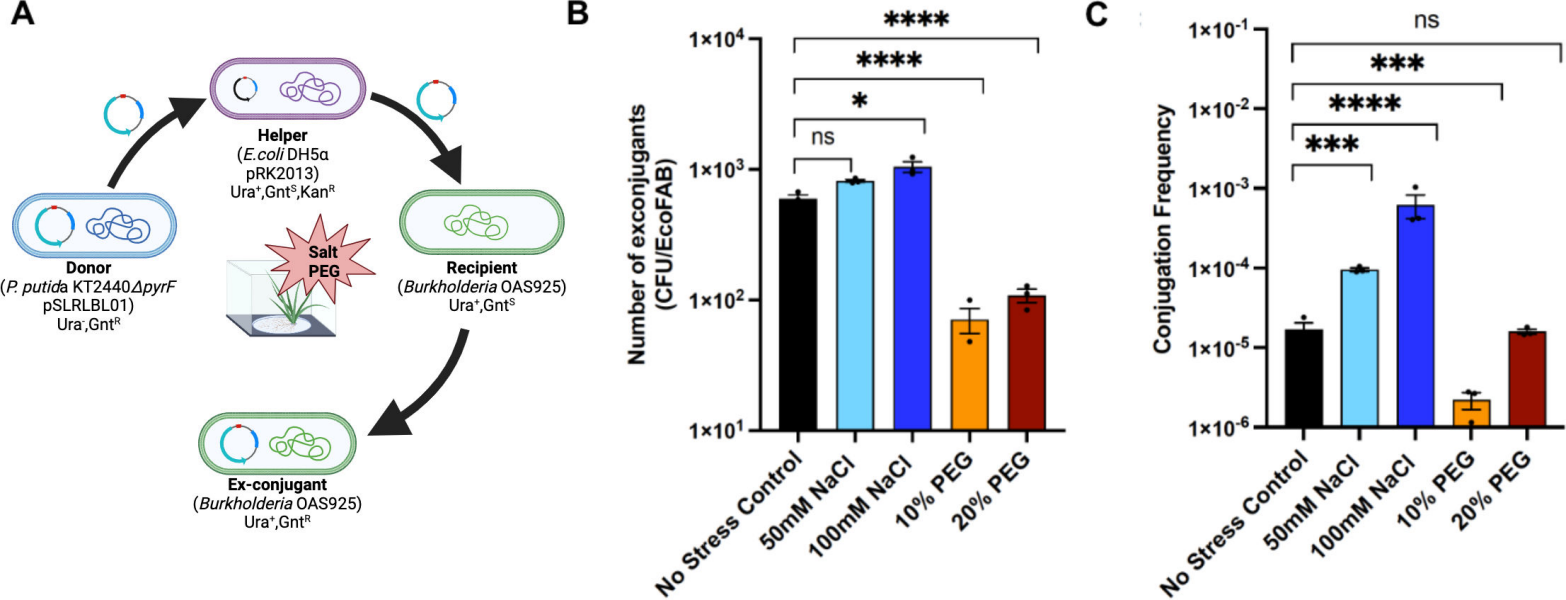
Schematic of conjugation experiment in the EcoFABs (**A**), the number of exconjugants (CFU per EcoFAB) (**B**), and the conjugation frequency (**C**) observed when *Burkholderia* sp. OAS925 was used as the recipient strain. The error bars show the mean standard error of the three replicates.

Our results indicate that *Burkholderia* sp. OAS925 can also take up the plasmid from another bacterial genus efficiently, and the frequency of conjugation increases under stressful environmental conditions such as salinity. We observed that under normal (no-stress) conditions, the number of exconjugants in the rhizosphere was ~600 CFU/EcoFAB when OAS925 was the recipient and *P. putida* was the donor of the plasmid, and it was ~20× lower than observed with the recipient of the same species (Fig. 5A and 6A). Addition of salt in the medium increased the intergenera transfer of plasmids, and the highest frequency of 6 × 10^−4^ was observed in the rhizosphere supplemented with 100-mM salt (Fig. 6B). The cell densities of the two bacterial genera in the salt-added media were lower than under the no-stress conditions with both high and low NaCl levels, indicating a negative impact of salt on the growth of both recipient and donor cells (Fig. S5). This shows that stress due to high salt concentrations could trigger plasmid transfer between different bacterial genera in the rhizosphere.

Increase in osmotic levels by adding PEG to the medium lowered the number of exconjugants and conjugation frequency. Nevertheless, while the density of donor and recipient decreased at the higher concentration of PEG (20%), it was comparable to the no stress conditions under the lower PEG (10%) concentration (Fig. S5). This suggests that the final HGT frequency under these conditions is potentially a trade-off between the number of cells and the stress that may induce gene transfer.

These results provide evidence of possible transmission of plasmids between two different bacterial genera in the rhizosphere microbial community under both normal and abiotic stress conditions. It has been shown previously that bacteria of different genera demonstrated plasmid transfer to improve survival in stress conditions (18, 44); however, it has not been extensively studied in the rhizosphere. Additionally, different types of stresses may have different levels of impact on HGT between different bacterial species, and the possible mechanisms involved are not fully characterized. Further studies will reveal the fate of plasmids in the presence of a complex rhizosphere microbial community mimicking the natural conditions of the rhizosphere and which factors may influence the frequency of HGT.

### Influence of helper plasmids on HGT frequency

In natural environments, HGT via conjugation usually involves a donor carrying a self-transmissible conjugative plasmid that is transferred to another bacteria (the recipient) in the vicinity. These plasmids often have a transfer (*tra*) gene cluster which encodes all the proteins required for their efficient transfer from donor to recipient (22). In some cases, including this study, the donor strains may not have this gene cluster and contain non-self-transmissible mobilizable plasmids. In such cases, these helper plasmids may facilitate the transfer of non-conjugative mobilizable plasmids to the recipients (represented by triparental conjugation) (45). Exchange of genetic material between two strains is more commonly documented, and the majority of the studies on horizontal gene transfer via conjugation involves self-transmissible conjugative plasmids (10, 22, 27). It is less understood if transfer of a non-self-transmissible plasmid is possible if there is no helper strain available and if the presence of natural environmental stress can stimulate this process. The donor plasmid (pSLRLBL01) used in this study is a mobilizable plasmid and does not have the *tra* gene cluster and therefore is a non-self-transmissible plasmid. Additionally, we investigated for the presence of origin of transfer (oriT) in the donor plasmid by annotating it using the established databases such as oriTfinder (46). Our analysis confirmed that the plasmid lacks the oriT sequences (see supplemental information). To examine the possibility of plasmid transfer without a helper strain, we performed biparental mating (as described in “Conjugation experiment on the LB agar plates,” below) on the LB-uracil medium using only the *P. putida* donor and recipient strains.

**Fig 7:**
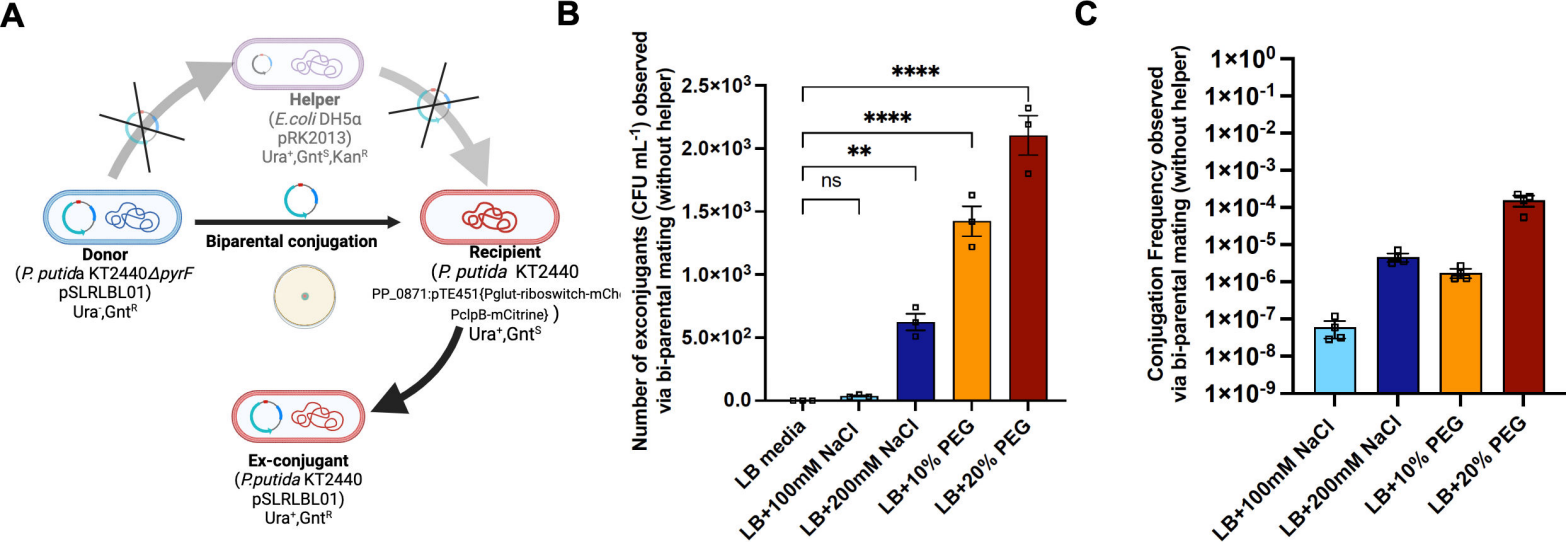
Schematic of biparental conjugation experiment (**A**), the number of exconjugants (CFU per mL) (**B**), and the conjugation frequency (**C**) observed via biparental mating on LB medium under salinity and osmotic stress conditions. The error bars show the mean standard error of the three replicates.

No exconjugants were observed on LB-uracil medium plates under routine growth conditions. However, addition of salt or PEG in the medium led to detectable plasmid transfer. The highest frequency observed was ~10^−4^ in the LB medium with 20% PEG (Fig. 7C). This frequency is several orders of magnitude lower than that observed in the triparental mating condition on LB-uracil medium agar plates that contain the helper *Escherichia coli* strain (Fig. 3B). This experiment was repeated several times and led to repeatable results. The control experiment conducted using only recipient strains with purified plasmid DNA did not show any plasmid uptake. This experiment was also conducted in the EcoFABs with *P. putida* as donor and recipient inoculated in the rhizosphere of *Brachypodium* under no-stress (0.5× MS media) and stress (0.5× MS media added with salt and PEG) conditions. However, in this case, no exconjugants were observed in the rhizosphere environment, likely due to the overall decrease in efficiency between HGT compared to the agar plate setup and the soil environment. While we ruled out the possibility of natural transformation in this case, substantial additional experiments are required to further examine the observed plasmid transfer in the absence of a known conjugative helper system and if this can be observed in a plant root-associated environment. Environmental stressors alone may enhance HGT rates to detectable levels without a strict requirement for a helper strain. These observations indicate that it may be useful to also examine HGT frequencies in microbial communities that may not have self-transmissible plasmids and under a range of plant root conditions and environmental stresses.

### Conclusions

EcoFABs provide an efficient and useful platform for assessing HGT frequency in the rhizosphere as they can more closely simulate the highly complex conditions in a rhizosphere environment. We observed plasmid transfer under both normal and stressful environmental conditions and investigated different parameters that may influence this process. The ratio of donor to recipient cells had a major impact on the conjugation frequency and may vary due to the difference in the fitness of the donor and recipient bacteria. The developmental stage of the plant and the root exudate composition also influence this frequency. We have also shown the possibility of conjugation between *P. putida* and *Burkholderia* sp. OAS925, providing evidence that some of the commonly found bacteria in the rhizosphere can efficiently take up plasmids from other microbial genera. In addition, we observed that intraspecies plasmid transfer is increased in the conditions of moderate osmotic stress, while saline conditions facilitate the conjugation between bacteria of different genera. This study underscores the multi-parametric nature of the rhizosphere with dynamic enhancing and inhibiting conditions that can impact HGT. Therefore, a controlled way to modulate multiple factors to thoroughly investigate the gene transfer process is required for which EcoFABs could be a very useful tool. This study provides sufficient evidence to confirm the HGT process under various conditions in the rhizosphere that can be quantified using EcoFABs. Therefore, precautionary measures are needed when disseminating engineered microbes containing mobilizable plasmids in the field or in agricultural environments.

## MATERIALS AND METHODS

### Growing plants in EcoFABs

All plant experiments were performed with *B. distachyon* Bd21-3 as the host plant (47). Seeds were dehusked and sterilized in 70% vol/vol ethanol for 30 s and in 6% vol/vol sodium hypochlorite for 5 min, followed by five wash steps in sterilized water, and kept at 4°C under dark conditions for stratification for 7 days. Seedlings were germinated on 0.5× MS plates [2.2 g/L MS medium, MSP01 (Caisson Labs, Smithfield, UT, USA) with 1,650 mg/L ammonium nitrate, 6.2 mg/L boric acid, 332.2 mg/L calcium chloride, 0.025 mg/L cobalt chloride, 0.025 mg/L copper sulfate, 37.26 mg/L disodium EDTA, 27.8 mg/L ferrous sulfate heptahydrate, 180.7 mg/L magnesium sulfate, 16.9 mg/L manganese(II) sulfate monohydrate, 0.25 g/L sodium molybdate dihydrate, 0.83 mg/L potassium iodide, 1,900 mg/L potassium nitrate, 170 mg/L monopotassium phosphate, and 8.6 mg/L zinc sulfate heptahydrate; 6% wt/vol phytoagar (Fisher Scientific, Waltham, MA, USA; pH adjusted to 5.8) in a 16 h:8 h, light:dark regime at 24°C with 150 µmol/m^2^/s illumination in reach-in plant growth chambers (Controlled Environments Inc., Pembina, ND, USA)] (48). EcoFAB 2.0 (6) was sterilized, and seedlings were transferred to EcoFAB chambers 3 days after germination. The EcoFAB root chambers were filled with 10 g of sand (sand, 50–70 mesh particle size; Sigma-Aldrich, St. Louis, MO, USA) and 5 mL of 0.5× MS before transferring to a humidity-controlled growth chamber. EcoFABs with plants were incubated in the plant growth chambers under the same conditions as mentioned above for seed germination. To impose osmotic stress conditions, 0.5× MS was added with 10% and 20% polyethylene glycol (PEG 6000, Sigma-Aldrich) for low and high stress levels, respectively, before adding the media into the EcoFABs (49). For salinity stress, 0.5× MS was supplemented with 50- or 100-mM NaCl (Sigma-Aldrich).

### Harvesting and collection of exconjugants from the rhizosphere

Plants were harvested 10, 20, and 30 DAG for root exudate collection for high-performance liquid chromatography (HPLC) (see “Measuring impact of donor-to-recipient ratio on conjugation frequency in the rhizosphere,” above) and to examine the effect of plant developmental stages on conjugation frequency. For all other experiments, plants were harvested after 2 weeks of growth (~5 primary leaves under optimum conditions) and 24 h post-inoculation. To examine if the presence of roots influences the conjugation frequency, we removed the roots from three EcoFABs (2-week-old plants) using sterile forceps after unscrewing the EcoFAB from top under aseptic conditions and dissolved the roots in 1-mL sterile water, vortexed for 10 min to dissolve all the root exudates attached to the roots; this slurry was added back into the same EcoFABs and mixed well using a sterile spatula for uniform distribution of root exudates. All other EcoFABs for this set of experiments (see “Growing plants in EcoFABs,” above) were also watered with 1-mL sterile water simultaneously.

All EcoFABs (except those grown for root exudate collection and HPLC analysis) were then inoculated and incubated for 24 h (see “Detection of HGT between bacteria of different genera in the rhizosphere,” above) before harvesting day. The incubated EcoFABs were then opened under aseptic conditions to extract the transconjugants. The EcoFABs containing inoculated *Brachypodium* were unscrewed from the top; the roots were cut from the shoots using sterile blades and pulled out using sterile tweezers; and the sand, along with the roots (rhizosphere), was transferred to 50-mL sterile centrifuge tubes containing 1-mL 1× phosphate-buffered saline (PBS). The tubes containing the roots in the PBS were shaken for 10 min to separate the attached microbes from the rhizosphere into suspension. The suspension was then pipetted into a microcentrifuge tube, serially diluted, and plated as detailed in the next section.

### Extraction of root exudates and quantification of sugars and organic acids

For root exudate collection, plants were grown separately for 10, 20, and 30 DAG in replicates in the EcoFABs following the same procedure as in “Detection and quantification of horizontal gene transfer via conjugation in the rhizosphere of *B. distachyon*,” above. EcoFABs filled with 10 g of sand (no plant) and 5 mL of 0.5× MS were used as controls. The sand, along with the roots (without roots for controls), was separated from shoots and transferred into 50-mL sterile centrifuge tubes containing sterile 10 mL of sterile ultrapure water and was gently stirred for 30 min. The sand settled down; the supernatant was filter-sterilized using a 0.22-µm filter and lyophilized. The freeze-dried root exudates were dissolved in 500 µL of sterile HPLC grade water and stored at −20°C for downstream applications. The amounts of sugars and organic acids in the root exudates were quantified using the Agilent HPLC 1260 infinity system (Santa Clara, CA, USA) equipped with a Bio-Rad Aminex HPX-87H column and a refractive index detector. An aqueous solution of sulfuric acid (4 mM) was used as the eluent (0.6 mL/min, column temperature of 60°C).

### Strains and plasmids used in the study

The uracil auxotroph *Pseudomonas putida* KT2440 Δ*pyrF* harboring the plasmid pSRLBL01 was used as a plasmid donor. This plasmid was used as a proxy for any commonly used engineered plasmid and carried genes that are the backbone of developing engineered microorganisms. The donor plasmid contains genes for gentamicin resistance as well as *cfp* encoding the cyan fluorescent protein. This plasmid was derived from the pTE252 (pBAD-sfp-bpsA GNT BBR1) plasmid used in a previous study (50) by replacing the *bpsA* gene with *cfp*. The PCR amplified product of the *cfp* gene was introduced into linearized pTE252 vector using NEBuilder HiFi DNA Assembly Cloning kit (New England Biolabs, Ipswich, MA, USA). A schematic of the pSRLBL01 (donor plasmid) map is presented in Fig. S7B, and the plasmid DNA sequences are provided in the supplemental file. The recipient strain used was *P. putida* KT2440 with a chromosomally integrated mCherry gene. To improve the conjugation frequency of the mobilizable, non-self-transmissible donor plasmid, we included a helper strain, *E. coli* DH5ɑ, harboring the plasmid pRK2013 containing the *tra* genes that aides the transfer of donor plasmid to the recipient strain via a triparental conjugation. For the intergenera conjugation experiments, we used *Burkholderia* sp. OAS925 as the recipient strain for the same plasmid, pSRLBL01, with the donor being also *Pseudomonas putida* KT2440 Δ*pyrF*.

### Preparation of bacterial cultures for triparental conjugation

The recipient strain *P. putida* KT2440 was grown in LB broth, while *Burkholderia* sp. OAS925 was grown in Reasoner’s 2A broth (51), and the donor strain was grown in LB broth supplemented with 0.2-μm filter-sterilized 25 µg/m uracil (Sigma-Aldrich) and 30 mg/L gentamicin. All the strains were incubated overnight at 30°C with shaking at 200 RPM on an orbital shaker. The helper strain (*E. coli* DH5ɑ) was grown in LB broth containing 50-mg/L kanamycin and incubated overnight at 37°C and 200 RPM. The bacterial cells grown overnight were harvested by centrifuging at 4,000 × *g* for 10 min and washed twice with sterile PBS. The cell pellet was resuspended in sterile PBS, and the OD_600_ was adjusted to obtain ~10^5^ CFU/mL of each strain (OD_600_ ~0.01 for recipient and donor and ~0.001 for helper).

### Conjugation experiment in EcoFABs

To determine the conjugation frequency in the rhizosphere, *B. distachyon* plants grown in the EcoFABs (see “Detection and quantification of horizontal gene transfer via conjugation in the rhizosphere of *B. distachyon*,” above) were inoculated with the mixture of all three strains. Bacterial donor, recipient, and helper cells were mixed in a microcentrifuge tube in the ratio of 10:1:1 to a total volume of 100 µL. To evaluate the impact of donor inoculum level on the conjugation frequency, one set of plants was also inoculated with the mixture of donor to recipient to helper in the ratios of 1:1:1 and 100:1:1. For environmental stress-based experiments (see “Strains and plasmids used in the study,” above), the ratio used was 50:1:1 to compensate for further inhibition of donor cell growth from the imposed stress by salt and PEG. The inoculated plants were moved to the growth chamber, and the bacterial cells were allowed to grow and conjugate for 24 h under the same conditions the plants were grown previously. The workflow for this experiment is shown in Fig. 2.

### Conjugation experiment on the LB agar plates

To compare the conjugation frequency in nutrient dynamic conditions of the rhizosphere with the optimum nutrient conditions for bacteria on rich media, a triparental mating was performed on LB agar plates supplemented with 25 µg/mL of uracil. For conjugation on plates, donor, recipient, and helper cells were mixed in a microcentrifuge tube in the ratio of 10:1:1 with the total volume of 100 µL and were plated on LB-uracil plates. The plates were then incubated at 30°C for 24 h. For the DNA transfer experiment without any helper strain, only the *P. putida* KT2440 donor and *P. putida* recipient strains (see Table 1) were mixed in a centrifuge tube at the ratio of 10:1 and plated on LB plates with uracil. This represents a form of biparental mating and is also shown in Fig. 7A. We define this method as “biparental” because this is the conventional method used for the conjugation between self-transmissible (tra^+^) donor and recipient strains. To understand the impact of external or environmental stress on this form of biparental mating, the mixed cultures of strains were also plated on LB-uracil plates with 100-mM NaCl and 200-mM NaCl, or with 10% PEG and 20% PEG.

**TABLE 1:** Plant, bacterial strains, and plasmids used in the study

Plant, strain, or plasmid	Genotype or phenotype	Reference
*Brachypodium distachyon* BD21-3	Plant	(47)
*Pseudomonas putida* KT2440 Δ*pyrF* (JBEI-204817)	Donor strain and uracil auxotroph	(52)
*P. putida* KT2440 PP_0871:pTE451 (Pglut-riboswitch-mCherry PclpB-mCitrine) Kan^S^ Suc^R^ (JBEI-255664)	Recipient strain	This study
*E. coli* DH5ɑ (pRK2013)	Tra^+^ Mob^+^ (RK2) Km::Tn*7* ColEl origin, helper plasmid, Km^R^	(53)
*Burkholderia* sp. OAS925	Recipient strain	(54)
pSLRLBL01 (JBEI-255655)	Donor plasmid Gnt^R^, pBAD *cfp*	This study
p101mNeonGreen (JBEI-255460)	Substitution of donor plasmid with mNeonGreen fluorescent genes for imaging, Kan^R^, pGingerBK-J23101 *mNeonGreen*	(55)

To eliminate the possibility of plasmid transfer via natural uptake/transformation, we also performed the experiment with only the recipient strain (*P. putida* KT2440 with genomically integrated mCherry) and a solution of purified donor plasmid (pSRLBL01) DNA. On LB plates (supplemented with salt/PEG for stress), we added 100 µL of the recipient mixed with 1, 10, or 100 µg/mL of plasmid, respectively, and incubated the plates for 24 h at 30°C to observe any colony formation.

### Detection and enumeration of recipients, donors, and exconjugants

After 24 h of incubation, 1 mL of bacterial suspensions from the plant rhizosphere in the EcoFABs was collected as described above (see “Impact of root exudates and plant developmental stages on frequency of HGT in the rhizosphere,” above) and serially diluted using PBS. For the conjugation on LB-uracil medium plates, the conjugants were scraped from the plates using a sterile inoculation loop, dissolved in 1-mL PBS, and serially diluted.

For each dilution, 100 µL was spread onto M9 agar plates; M9 agar plates were supplemented with gentamicin and with 25 µg/L of uracil. The number of recipients was counted as the number of colonies on M9 agar plates; the number of transconjugants was counted as the number of colonies on M9 agar plates supplemented with gentamicin; and the number of donors was counted by subtracting the number of transconjugants from the total number of colonies in the M9 agar plate supplemented with uracil and gentamicin. The conjugation frequency was calculated as mentioned below.

The CF was calculated by referring to the equation CF = NC/NR, where NC indicates the CFU/mL (for agar plate) or CFU/EcoFAB (for rhizosphere) of the exconjugants, and NR represents the CFU/mL or CFU/EcoFAB of recipients (17).

### Graphical representation and statistical analysis of data

GraphPad Prism 9 (GraphPad Software, San Diego, CA, USA) was used to make all graphical figures and perform statistical analysis. Significant differences were assessed using Student’s *t*-test and one-way analysis of variance on log10-transformed data (56). At a 95% confidence interval, a *P* value of ≥0.05 was considered insignificant. In the figures, **** indicates very high statistical significance (*P* < 0.0001); *** indicates high statistical significance (*P* < 0.001); ** indicates high statistical significance (*P* < 0.01); * indicates statistical significance (*P* < 0.05); and ns indicates non-significance.

### Microscope imaging of plant roots

The images of the inoculated roots were taken with an EVOS M7000 fluorescence microscope (Thermo Fisher Scientific) using objectives with ×2 and ×40 magnification. For imaging purposes, we replaced the plasmid in the donor strain with p101mNeonGreen (55), which constitutively expressed neon green fluorescence. Both donor (green) and recipient (mCherry red) were inoculated in the *B. distachyon* rhizosphere and grown for 2 weeks, and images were taken 24 h post-inoculation. To accommodate for color vision deficiency, we switched the red color of mCherry to magenta using the Fiji software package for ImageJ (57). The original images without these changes are shown in Fig. S6.

## SUPPLEMENTAL MATERIAL

Supplemental material aem.01505-24-s0001.pdf Figures S1 to S7; supplemental methods.

Supplemental text aem.01505-24-s0002.txt DNA sequence of pSLRLBL01.

ASM does not own the copyrights to Supplemental Material that may be linked to, or accessed through, an article. The authors have granted ASM a non-exclusive, world-wide license to publish the Supplemental Material files. Please contact the corresponding author directly for reuse.

## References

[1] Frost LS, Leplae R, Summers AO, Toussaint A. 2005. Mobile genetic elements: the agents of open source evolution. Nat Rev Microbiol 3:722–732. doi:10.1038/nrmicro1235 16138100

[2] Wiedenbeck J, Cohan FM. 2011. Origins of bacterial diversity through horizontal genetic transfer and adaptation to new ecological niches. FEMS Microbiol Rev 35:957–976. doi:10.1111/j.1574-6976.2011.00292.x 21711367

[3] Smit E, Elsas JD. 1992. Conjugal gene transfer in the soil environment; new approaches and developments, p 79–94. In Gauthier MJ (ed), Gene transfers and environment. Springer, Berlin Heidelberg, Berlin, Heidelberg.

[4] Heuer H, Smalla K. 2007. Horizontal gene transfer between bacteria. Environ Biosafety Res 6:3–13. doi:10.1051/ebr:2007034 17961477

[5] Yee MO, Kim P, Li Y, Singh AK, Northen TR, Chakraborty R. 2021. Specialized plant growth chamber designs to study complex rhizosphere interactions. Front Microbiol 12:625752. doi:10.3389/fmicb.2021.625752 33841353 PMC8032546

[6] Novak V, Andeer PF, Bowen BP, Ding Y, Zhalnina K, Hofmockel KS, Tomaka C, Harwood TV, van Winden MCM, Golini AN, Kosina SM, Northen TR. 2024. Reproducible growth of Brachypodium in EcoFAB 2.0 reveals that nitrogen form and starvation modulate root exudation. Sci Adv 10:eadg7888. doi:10.1126/sciadv.adg7888 38170767 PMC10776018

[7] Gao J, Sasse J, Lewald KM, Zhalnina K, Cornmesser LT, Duncombe TA, Yoshikuni Y, Vogel JP, Firestone MK, Northen TR. 2018. Ecosystem Fabrication (EcoFAB) protocols for the construction of laboratory ecosystems designed to study plant-microbe Interactions. JoVE e57170:57170. doi:10.3791/57170 PMC5933423 29708529

[8] Sasse J, Kant J, Cole BJ, Klein AP, Arsova B, Schlaepfer P, Gao J, Lewald K, Zhalnina K, Kosina S, Bowen BP, Treen D, Vogel J, Visel A, Watt M, Dangl JL, Northen TR. 2019. Multilab EcoFAB study shows highly reproducible physiology and depletion of soil metabolites by a model grass. New Phytol 222:1149–1160. doi:10.1111/nph.15662 30585637 PMC6519027

[9] Lin H-H, Torres M, Adams CA, Andeer PF, Owens TK, Zhalnina K, Jabusch LK, Carlson HK, Kuehl JV, Deutschbauer AM, Northen TR, Glass NL, Mortimer JC. 2023. Impact of inoculation practices on microbiota assembly and community stability in a fabricated ecosystem. Plant Biology. doi:10.1101/2023.06.13.544848

[10] Mølbak L, Molin S, Kroer N. 2007. Root growth and exudate production define the frequency of horizontal plasmid transfer in the Rhizosphere. FEMS Microbiol Ecol 59:167–176. doi:10.1111/j.1574-6941.2006.00229.x 17069619

[11] Kroer N, Barkay T, SÃ¸rensen S, Weber D. 1998. Effect of root exudates and bacterial metabolic activity on conjugal gene transfer in the rhizosphere of a marsh plant. FEMS Microbiol Ecol 25:375–384. doi:10.1111/j.1574-6941.1998.tb00489.x

[12] Maheshwari M, Abulreesh HH, Khan MS, Ahmad I, Pichtel J. 2017. Horizontal gene transfer in soil and the rhizosphere: impact on ecological fitness of bacteria, p 111–130. In Meena VS, Mishra PK, Bisht JK, Pattanayak A (ed), Agriculturally important microbes for sustainable agriculture. Springer Singapore, Singapore.

[13] Hacker J, Kaper JB. 2000. Pathogenicity islands and the evolution of microbes. Annu Rev Microbiol 54:641–679. doi:10.1146/annurev.micro.54.1.641 11018140

[14] Beuls E, Modrie P, Deserranno C, Mahillon J. 2012. High-salt stress conditions increase the pAW63 transfer frequency in Bacillus thuringiensis. Appl Environ Microbiol 78:7128–7131. doi:10.1128/AEM.01105-12 22820331 PMC3457476

[15] Li L-G, Zhang T. 2023. Plasmid-mediated antibiotic resistance gene transfer under environmental stresses: Insights from laboratory-based studies. Sci Total Environ 887:163870. doi:10.1016/j.scitotenv.2023.163870 37149187

[16] Richaume A, Angle JS, Sadowsky MJ. 1989. Influence of soil variables on in situ plasmid transfer from Escherichia coli to Rhizobium fredii. Appl Environ Microbiol 55:1730–1734. doi:10.1128/aem.55.7.1730-1734.1989 2669634 PMC202942

[17] Shi H, Hu X, Xu J, Hu B, Ma L, Lou L. 2023. Conjugation-mediated transfer of antibiotic resistance genes influenced by primary soil components and underlying mechanisms. Sci Total Environ 865:161232. doi:10.1016/j.scitotenv.2022.161232 36586689

[18] Zhang S, Wang Y, Song H, Lu J, Yuan Z, Guo J. 2019. Copper nanoparticles and copper ions promote horizontal transfer of plasmid-mediated multi-antibiotic resistance genes across bacterial genera. Environ Int 129:478–487. doi:10.1016/j.envint.2019.05.054 31158594

[19] Argyle JL, Rapp-Giles BJ, Wall JD. 1992. Plasmid transfer by conjugation in Desulfovibrio desulfuricans. FEMS Microbiol Lett 73:255–262. doi:10.1016/0378-1097(92)90640-a 1426989

[20] Billi D, Friedmann EI, Helm RF, Potts M. 2001. Gene transfer to the desiccation-tolerant cyanobacterium Chroococcidiopsis. J Bacteriol 183:2298–2305. doi:10.1128/JB.183.7.2298-2305.2001 11244070 PMC95137

[21] Kluepfel DA. 1993. The behavior and tracking of bacteria in the rhizosphere. Annu Rev Phytopathol 31:441–472. doi:10.1146/annurev.py.31.090193.002301

[22] Elsas JD, Trevors JT, Starodub ME. 1988. Bacterial conjugation between pseudomonads in the rhizosphere of wheat. FEMS Microbiol Lett 53:299–306. doi:10.1111/j.1574-6968.1988.tb02676.x-i1

[23] Vilas-Bôas LA, Vilas-Bôas GF, Saridakis HO, Lemos MV, Lereclus D, Arantes OM. 2000. Survival and conjugation of Bacillus thuringiensis in a soil microcosm. FEMS Microbiol Ecol 31:255–259. doi:10.1111/j.1574-6941.2000.tb00691.x 10719207

[24] Lafuente R, Maymó-Gatell X, Mas-Castellà J, Guerrero R. 1996. Influence of environmental factors on plasmid transfer in soil microcosms. Curr Microbiol 32:213–220. doi:10.1007/s002849900038

[25] Fan X-T, Li H, Chen Q-L, Zhang Y-S, Ye J, Zhu Y-G, Su J-Q. 2019. Fate of antibiotic resistant pseudomonas putida and broad host range plasmid in natural soil microcosms. Front Microbiol 10:194. doi:10.3389/fmicb.2019.00194 30881351 PMC6407330

[26] Macedo G, Olesen AK, Maccario L, Hernandez Leal L, V D Maas P, Heederik D, Mevius D, Sørensen SJ, Schmitt H. 2022. Horizontal gene transfer of an incp1 plasmid to soil bacterial community introduced by escherichia coli through manure amendment in soil microcosms. Environ Sci Technol 56:11398–11408. doi:10.1021/acs.est.2c02686 35896060 PMC9387108

[27] Schwaner NE, Kroer N. 2001. Effect of plant species on the kinetics of conjugal transfer in the rhizosphere and relation to bacterial metabolic activity. Microb Ecol 42:458–465. doi:10.1007/s00248-001-0001-4 12024270

[28] Vora SM, Joshi P, Belwalkar M, Archana G. 2021. Root exudates influence chemotaxis and colonization of diverse plant growth promoting rhizobacteria in the pigeon pea – maize intercropping system. Rhizosphere 18:100331. doi:10.1016/j.rhisph.2021.100331

[29] Aulakh MS, Wassmann R, Bueno C, Kreuzwieser J, Rennenberg H. 2001. Characterization of root exudates at different growth stages of ten rice (Oryza sativa l.) cultivars . Plant Biol (Stuttg) 3:139–148. doi:10.1055/s-2001-12905

[30] Kawasaki A, Donn S, Ryan PR, Mathesius U, Devilla R, Jones A, Watt M. 2016. Microbiome and exudates of the root and rhizosphere of Brachypodium distachyon, a model for wheat.. PLoS ONE 11:e0164533. doi:10.1371/journal.pone.0164533 27727301 PMC5058512

[31] Yuan J, Zhang N, Huang Q, Raza W, Li R, Vivanco JM, Shen Q. 2015. Organic acids from root exudates of banana help root colonization of PGPR strain Bacillus amyloliquefaciens NJN-6. Sci Rep 5:13438. doi:10.1038/srep13438 26299781 PMC4547103

[32] Saleh D, Sharma M, Seguin P, Jabaji S. 2020. Organic acids and root exudates of Brachypodium distachyon: effects on chemotaxis and biofilm formation of endophytic bacteria. Can J Microbiol 66:562–575. doi:10.1139/cjm-2020-0041 32348684

[33] Xiong Y-W, Li X-W, Wang T-T, Gong Y, Zhang C-M, Xing K, Qin S. 2020. Root exudates-driven rhizosphere recruitment of the plant growth-promoting rhizobacterium Bacillus flexus KLBMP 4941 and its growth-promoting effect on the coastal halophyte Limonium sinense under salt stress. Ecotoxicol Environ Saf 194:110374. doi:10.1016/j.ecoenv.2020.110374 32120174

[34] Nielsen KM, van Elsas JD. 2001. Stimulatory effects of compounds present in the rhizosphere on natural transformation of acinetobacter sp. BD413 in soil. Soil Biol Biochem 33:345–357. doi:10.1016/S0038-0717(00)00147-4

[35] Lampkowska J, Feld L, Monaghan A, Toomey N, Schjørring S, Jacobsen B, van der Voet H, Andersen SR, Bolton D, Aarts H, Krogfelt KA, Wilcks A, Bardowski J. 2008. A standardized conjugation protocol to asses antibiotic resistance transfer between lactococcal species. Int J Food Microbiol 127:172–175. doi:10.1016/j.ijfoodmicro.2008.06.017 18675485

[36] Davies J, Spiegelman GB, Yim G. 2006. The world of subinhibitory antibiotic concentrations. Curr Opin Microbiol 9:445–453. doi:10.1016/j.mib.2006.08.006 16942902

[37] Molin S, Tolker-Nielsen T. 2003. Gene transfer occurs with enhanced efficiency in biofilms and induces enhanced stabilisation of the biofilm structure. Curr Opin Biotechnol 14:255–261. doi:10.1016/s0958-1669(03)00036-3 12849777

[38] Ortiz de la Rosa JM, Nordmann P, Poirel L. 2021. Antioxidant molecules as a source of mitigation of antibiotic resistance gene dissemination. Antimicrob Agents Chemother 65. doi:10.1128/AAC.02658-20 PMC8315946 33753335

[39] Kirk JA, Fagan RP. 2016. Heat shock increases conjugation efficiency in Clostridium difficile. Anaerobe 42:1–5. doi:10.1016/j.anaerobe.2016.06.009 27377776 PMC5154368

[40] Schäfer A, Kalinowski J, Pühler A. 1994. Increased fertility of Corynebacterium glutamicum recipients in intergeneric matings with Escherichia coli after stress exposure. Appl Environ Microbiol 60:756–759. doi:10.1128/aem.60.2.756-759.1994 8135527 PMC201381

[41] Gargallo-Garriga A, Preece C, Sardans J, Oravec M, Urban O, Peñuelas J. 2018. Root exudate metabolomes change under drought and show limited capacity for recovery. Sci Rep 8:12696. doi:10.1038/s41598-018-30150-0 30140025 PMC6107494

[42] Chai YN, Schachtman DP. 2022. Root exudates impact plant performance under abiotic stress. Trends Plant Sci 27:80–91. doi:10.1016/j.tplants.2021.08.003 34481715

[43] Schierstaedt J, Bziuk N, Kuzmanović N, Blau K, Smalla K, Jechalke S. 2019. Role of plasmids in plant-bacteria interactions. Curr Issues Mol Biol 30:17–38. doi:10.21775/cimb.030.017 30070649

[44] Zhang Y, Gu AZ, He M, Li D, Chen J. 2017. Subinhibitory concentrations of disinfectants promote the horizontal transfer of multidrug resistance genes within and across genera. Environ Sci Technol 51:570–580. doi:10.1021/acs.est.6b03132 27997135

[45] Zatyka M, Thomas CM. 1998. Control of genes for conjugative transfer of plasmids and other mobile elements. FEMS Microbiol Rev 21:291–319. doi:10.1111/j.1574-6976.1998.tb00355.x 25508777

[46] Li X, Xie Y, Liu M, Tai C, Sun J, Deng Z, Ou H-Y. 2018. oriTfinder: a web-based tool for the identification of origin of transfers in DNA sequences of bacterial mobile genetic elements. Nucleic Acids Res 46:W229–W234. doi:10.1093/nar/gky352 29733379 PMC6030822

[47] Vogel J, Hill T. 2008. High-efficiency Agrobacterium-mediated transformation of Brachypodium distachyon inbred line Bd21-3. Plant Cell Rep 27:471–478. doi:10.1007/s00299-007-0472-y 17999063

[48] Baudson C, Delory BM, Spaepen S, du Jardin P, Delaplace P. 2021. Developmental plasticity of Brachypodium distachyon in response to P deficiency: modulation by inoculation with phosphate-solubilizing bacteria. Plant Direct 5:e00296. doi:10.1002/pld3.296 33532689 PMC7833465

[49] O’Donnell NH, Møller BL, Neale AD, Hamill JD, Blomstedt CK, Gleadow RM. 2013. Effects of PEG-induced osmotic stress on growth and dhurrin levels of forage sorghum. Plant Physiol Biochem 73:83–92. doi:10.1016/j.plaphy.2013.09.001 24080394

[50] Gauttam R, Eng T, Zhao Z, Ul Ain Rana Q, Simmons BA, Yoshikuni Y, Mukhopadhyay A, Singer SW. 2023. Development of genetic tools for heterologous protein expression in a pentose-utilizing environmental isolate of Pseudomonas putida. Microb Biotechnol 16:645–661. doi:10.1111/1751-7915.14205 36691869 PMC9948227

[51] Reasoner DJ, Geldreich EE. 1985. A new medium for the enumeration and subculture of bacteria from potable water. Appl Environ Microbiol 49:1–7. doi:10.1128/aem.49.1.1-7.1985 3883894 PMC238333

[52] Czajka JJ, Banerjee D, Eng T, Menasalvas J, Yan C, Munoz NM, Poirier BC, Kim Y-M, Baker SE, Tang YJ, Mukhopadhyay A. 2022. Tuning a high performing multiplexed-CRISPRi Pseudomonas putida strain to further enhance indigoidine production. Metab Eng Commun 15:e00206. doi:10.1016/j.mec.2022.e00206 36158112 PMC9494242

[53] Figurski DH, Helinski DR. 1979. Replication of an origin-containing derivative of plasmid RK2 dependent on a plasmid function provided in trans. Proc Natl Acad Sci U S A 76:1648–1652. doi:10.1073/pnas.76.4.1648 377280 PMC383447

[54] Price MN, Deutschbauer AM, Arkin AP. 2022. Filling gaps in bacterial catabolic pathways with computation and high-throughput genetics. PLoS Genet 18:e1010156. doi:10.1371/journal.pgen.1010156 35417463 PMC9007349

[55] Pearson AN, Thompson MG, Kirkpatrick LD, Ho C, Vuu KM, Waldburger LM, Keasling JD, Shih PM. 2023. The pGinger family of expression plasmids. Microbiol Spectr 11:e0037323. doi:10.1128/spectrum.00373-23 37212656 PMC10269703

[56] St»hle L, Wold S. 1989. Analysis of variance (ANOVA). Chemometr Intell Lab Syst 6:259–272. doi:10.1016/0169-7439(89)80095-4

[57] Schindelin J, Arganda-Carreras I, Frise E, Kaynig V, Longair M, Pietzsch T, Preibisch S, Rueden C, Saalfeld S, Schmid B, Tinevez J-Y, White DJ, Hartenstein V, Eliceiri K, Tomancak P, Cardona A. 2012. Fiji: an open-source platform for biological-image analysis. Nat Methods 9:676–682. doi:10.1038/nmeth.2019 22743772 PMC3855844

