# Supplementary material for: Assessing horizontal gene transfer in the rhizosphere of Brachypodium distachyon using fabricated ecosystems (EcoFABs)
Source: Appl Environ Microbiol. 2024 Nov 4;90(11):e01505-24. doi: 10.1128/aem.01505-24 (PMC11577780; doi:10.1128/aem.01505-24)
Supplement: Supplemental material — Figures S1 to S7; supplemental methods. [file aem.01505-24-s0001.pdf]

Supplementary data

Assessing horizontal gene transfer in the rhizosphere of *Brachypodium distachyon* using fabricated ecosystems (EcoFABs)

Shweta Priya[1], Silvia Rossbach[3], Thomas Eng[1], Hsiao-Han Lin[2], Peter F. Andeer[2], Jenny C. Mortimer[1,2,4], Trent R. Northen[2], & Aindrila Mukhopadhyay[1,2*]

[1]Biological Systems and Engineering Division, Lawrence Berkeley National Laboratory, Berkeley, CA, 94720, USA

[2]Environmental Genomics and Systems Biology Division, Lawrence Berkeley National Laboratory, Berkeley, CA, 94720, USA

[3]Department of Biological Sciences, Western Michigan University, 1903 W Michigan Ave, Kalamazoo MI 49008-5410, U.S.A.

[4]School of Agriculture, Food and Wine, University of Adelaide, Australia

*correspondence: amukhopadhyay@lbl.gov

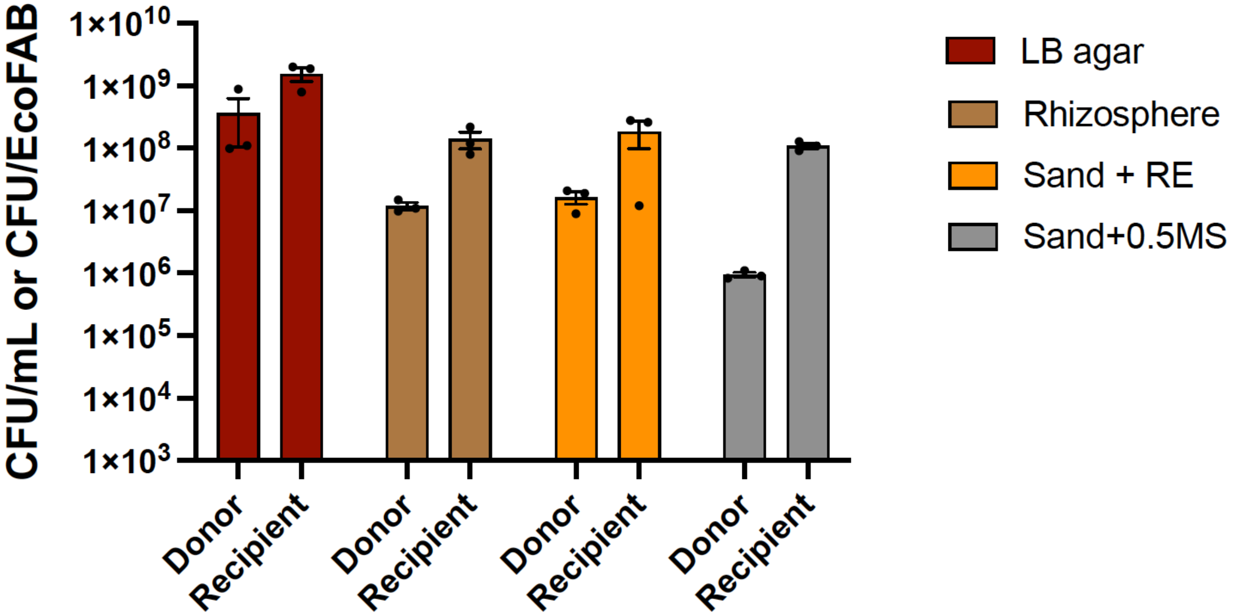

Fig S1. The number of donor and recipient (CFU per mL for LB-agar and CFU/EcoFAB for others) observed under different treatments. The error bars show the mean standard error of the three replicates.

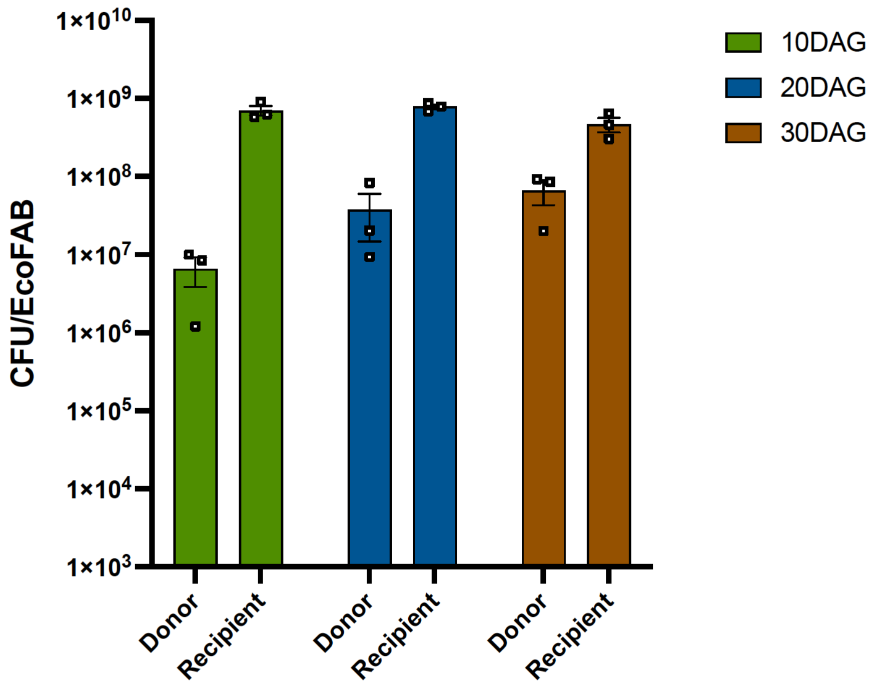

Fig S2. The number of donor and recipient (CFU per mL) observed at different developmental stages of the plant. DAG: days after germination. The error bars show the mean standard error of the three replicates.

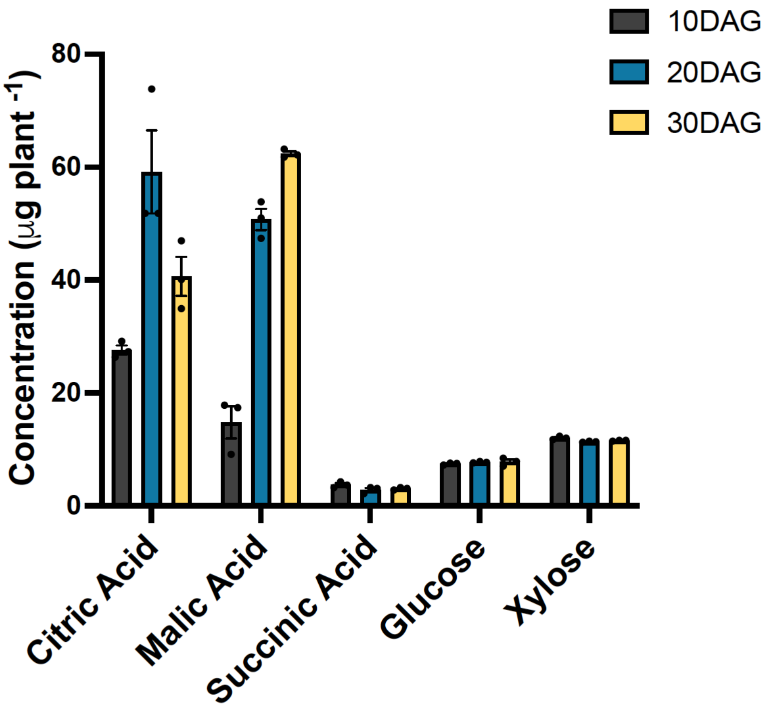

Fig S3. Concentration of organic acids and sugars detected in the root exudates extracted from BD21-3 at 10DAG, 20DAG and 30DAG. DAG: days after germination. The error bars show the mean standard error of the three replicates.

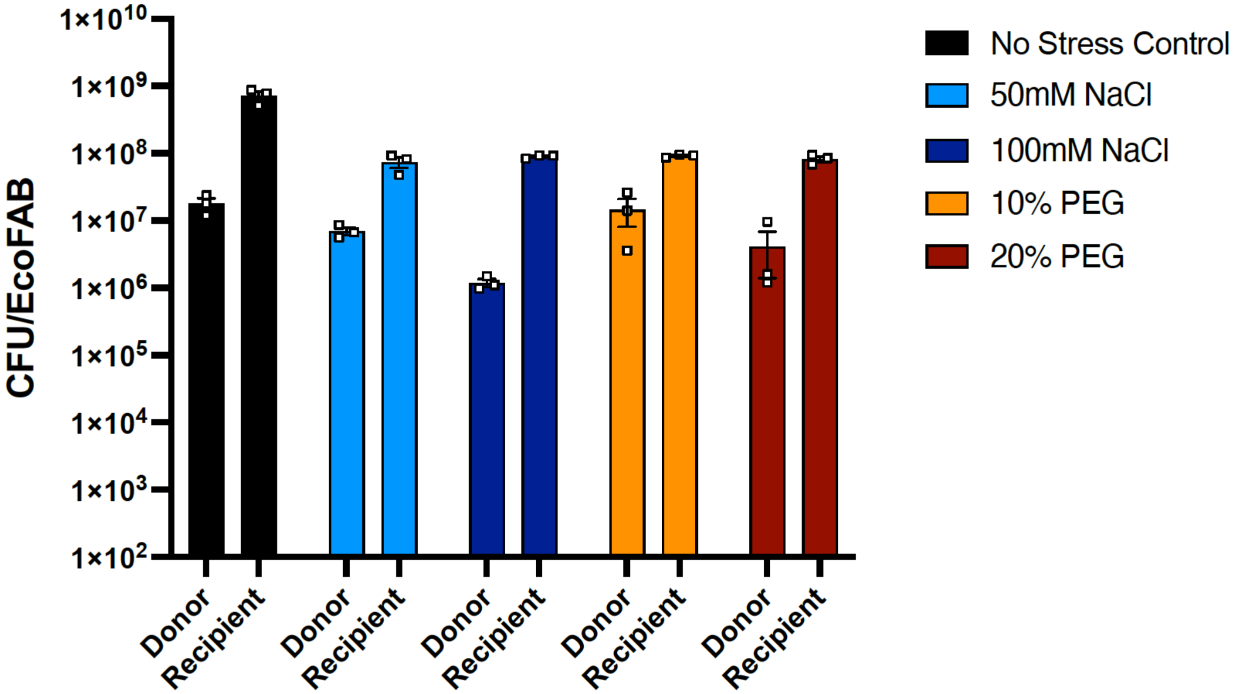

Fig S4. The number of donor and recipient (CFU per EcoFAB) observed under different treatments. The error bars show the mean standard error of the three replicates.

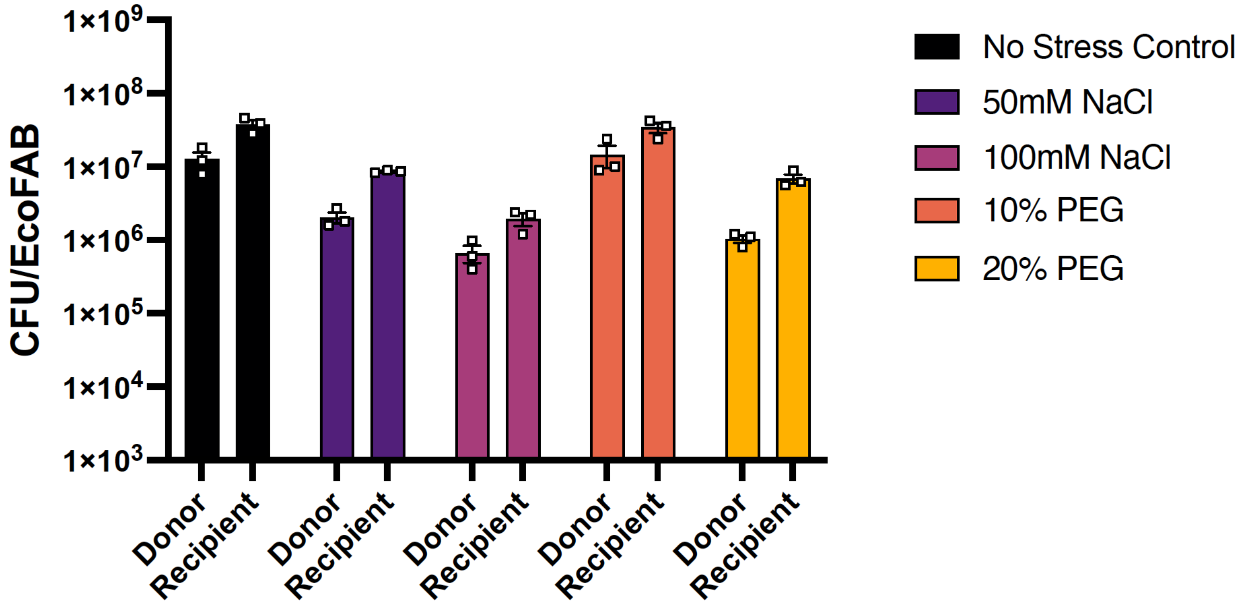

Fig S5. The number of donor (*P.putida* KT2440Δ*pyrF)* and recipient (*B. sp.* OAS925)(CFU per EcoFAB) observed under different treatments with intergeneric conjugation in the rhizosphere. The error bars show the mean standard error of the three replicates.

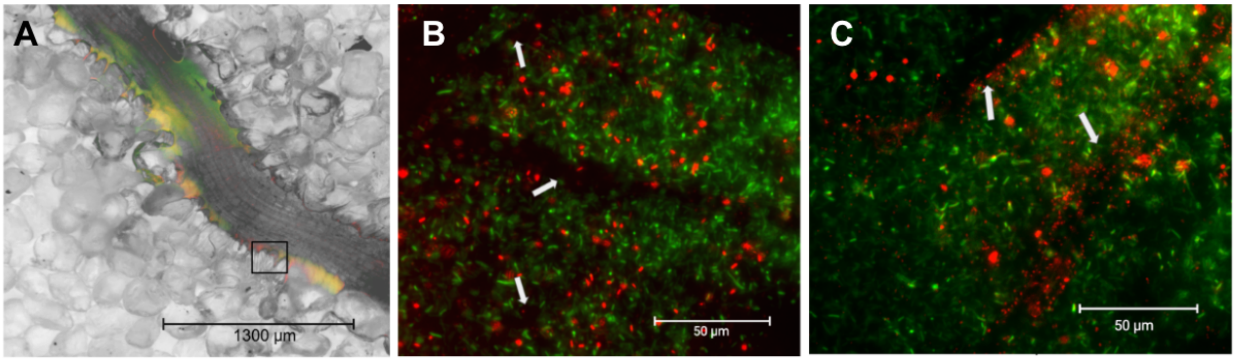

Fig S6. Microscopic images of *B. distachyon* roots inoculated with proxy donor (green) and recipient (red) strains in EcoFABs using 2X (A) and 40X (B, C) objectives. The black box in image A represents the area magnified for images B and C. White arrows in B and C represent the root hair. This is the original version of the image presented in Fig 3D-3F where red of mCherry is replaced by magenta color.

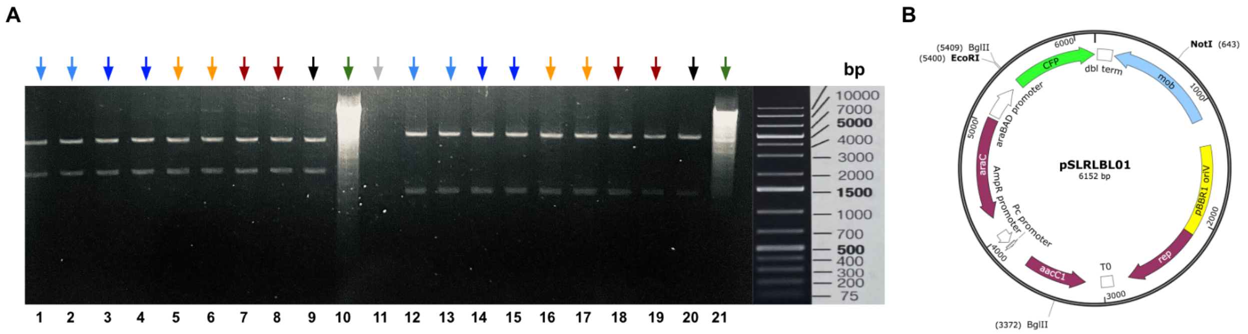

Fig S7: Gel image of restriction enzyme digestion of the plasmid (pSLRLBL01) isolated from the exconjugants obtained from HGT experiments without a helper strain (A). Lanes 1-10 are for the dual cutter BglII that cuts the plasmid into 4115 bp and 2037 bp fragments while lanes 12-21 are for NotI and EcoRI that cut the plasmid into 4757 bp and 1395 bp fragments. The different colored arrows represent different treatments: light blue : LB + 100mM Salt, dark blue: LB+200mM Salt, Orange: LB+ 10%PEG, brown : LB + 20%PEG, black : plasmid isolated from donor as positive control, green : genomic DNA of the recipient and gray : plasmid without restriction enzyme. Plasmid map for pSLRLBL01 showing the different restriction sites for all the restriction enzymes used (BglII, EcoRI and NotI and the major features of the plasmid (B).

Supplementary Information

Investigating the presence of origin of transfer (oriT) in the donor plasmid pSLRLBL01

In order to confirm the non-self-transmissible nature of the donor plasmid pSLRLBL01, we checked for the presence of sequences that represent origin of transfer (oriT) in a conjugative donor plasmid. We searched established databases to reannotate pSLRLBL01 in case it contained any known oriT sequences. After confirming the plasmid sequence by whole genome sequencing we examined the plasmid sequence again using Basic Local Alignment Search Tool (BLAST) based mechanisms, including oriTfinder [1], pLannotate [2] and known categorizations from the popular DNA sequence browser SnapGene (www.snapgene.com). The only instance that an oriT was detected was using the oriTfinder program, which suggested the sequence 5'-GTCACGACTTTGCGAAGCAAAGTCTAGTGAGTATACTCAAGCATTGAGTGGC-3' might be a potential new oriT sequence. However, no published evidence in NCBI's (BLAST) [3] supports this claim that would suggest this to be true. SnapGene indicates there are 3 known oriT sequences, which are:

1. incP oriT:

5'-
GGGCAGGATAGGTGAAGTAGGCCCACCCGCGAGCGGGTGTTCCTTCTTCACTGTCCC
TTATTCGCACCTGGCGGTGCTCAACGGGAATCCTGCTCTGCGAGGCTGGCCGG-3'

2. F plasmid oriT:

5'AAGGCTCAACAGGTTGGTGGTTCTCACCACCAAAAGCACCACACCCCACGCAAAA
ACAAGTTTTTGCTGATTTTTCTTTATAAATAGAGTGTTATGAAAAATTAGTTTCTCTT
ACTCTCTTTATGATATTTAAAAAAGCGGTGTCGGCGCGGCTACAACAACGCGCCGAC
ACCGTTTTGTAGGGGTGGTACTGACTATTTTTATAAAAAACATTATTTTATATTAGGG
GTGCTGCTAGCGGCGCGGTGTGTTTTTTTATAGGATACCGCTAGGGGCGCTGCTAGC
GGTGCG-3'

3. RSF1010 oriT:

5'-
TAGGCTATCATGGAGGCACAGCGGCGGCAATCCCGACCCTACTTTGTAGGGGAGGG
CGCACTTACCGGTTTCTCTTCGAGAAACTGG-3'

These sequences are not present in the donor plasmid. While the oriTfinder program may be accurate, confirming this would necessitate extensive testing. We prefer to rely on established

databases for known annotations to determine the presence of a compatible oriT sequence. Therefore, we can state with high confidence that the donor plasmid pSLRLBL101 used in this study does not contain oriT and therefore is not self-transmissible.

References

1 Li X, Xie Y, Liu M, Tai C, Sun J, Deng Z & Ou H-Y (2018) oriTfinder: a web-based tool for the identification of origin of transfers in DNA sequences of bacterial mobile genetic elements. *Nucleic Acids Res* **46**, W229–W234.

2 McGuffie MJ & Barrick JE (2021) pLannotate: engineered plasmid annotation. *Nucleic Acids Res* **49**, W516–W522.

3 Altschul SF, Gish W, Miller W, Myers EW & Lipman DJ (1990) Basic local alignment search tool. *J Mol Biol* **215**, 403–410.
